# 4-[4-(4-Chloro-1,2,5-thia­diazol-3-yl)phen­yl]morpholine

**DOI:** 10.1107/S2414314626004207

**Published:** 2026-05-07

**Authors:** Paul R. Palme, Richard Goddard, Adrian Richter, Peter Imming, Rüdiger W. Seidel

**Affiliations:** a Institut für Pharmazie, Martin-Luther-Universität Halle-Wittenberg, Wolfgang-Langenbeck-Str. 4, 06120 Halle (Saale), Germany; b Max-Planck-Institut für Kohlenforschung, Kaiser-Wilhelm-Platz 1, 45470 Mülheim an der Ruhr, Germany; Purdue University, USA

**Keywords:** crystal structure, Hirshfeld atom refinement, Hirshfeld surface analysis, thia­diazole, morpholine, Suzuki coupling

## Abstract

The title compound, C_12_H_12_ClN_3_OS, was prepared using a Suzuki–Miyaura cross-coupling reaction. The compound was found to crystallize in the ortho­rhom­bic system (space group *Pbca*, *Z* = 8). The crystal structure was refined with non-spherical atomic form factors using Hirshfeld atom refinement. The mean planes through the thia­diazole ring and the benzene ring are inclined at an angle of 36.83 (2)°. The morpholine ring adopts a chair conformation with a markedly pyramidal bonding situation at the N atom. The crystal packing is dense, with a packing index of 75%.

## Abstract 2

The crystal and mol­ecular structure of 4-[4-(4-chloro-1,2,5-thia­diazol-3-yl)phen­yl]morpholine are reported.

## Graphical abstract

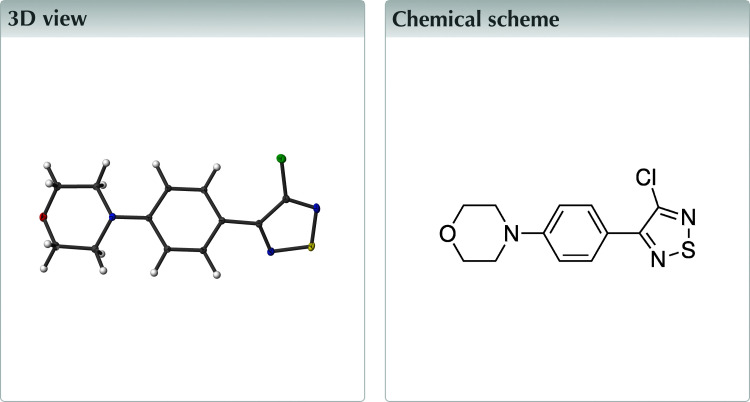

## Structure description

The 1,2,5-thia­diazole heterocyclic system has gained importance in medicinal chemistry, as well as agricultural and materials science (Quiroga *et al.*, 2025 ▸). The saturated six-membered morpholine heterocycle is part of many drug substances, adjusting the degree of polarity and ease of metabolism (Kumari & Singh, 2020 ▸). We prepared the title compound from (4-morpholino­phen­yl)boronic acid and 3,4-di­chloro-1,2,5-thia­diazole by a Suzuki–Miyaura heteroaryl cross-coupling reaction (Meringdal & Menche, 2025 ▸). It has been demonstrated previously that 3,4-di­chloro-1,2,5-thia­diazole undergoes Suzuki–Miyaura cross-coupling reactions to yield the corresponding mono-substituted derivatives, leaving one Cl atom for potential further functionalization (Merschaert & Gorissen, 2003 ▸). While the Cambridge Structural Database (CSD; Groom *et al.*, 2016 ▸) contains a wide variety of crystal structures of 4-phenyl­morpholine derivatives, a search using the *WebCSD* inter­face (Thomas *et al.*, 2010 ▸) in April 2026 revealed only one crystallographically characterized compound containing a 4-chloro-1,2,5-thia­diazol-3-yl group, namely, 2-(4-chloro-1,2,5-thia­diazol-3-yl)quinazolin-4(3*H*)-one (CSD refcode UQOGIT; Kalogirou *et al.*, 2021 ▸). The CSD entry DOCFEG features a 1,2,5-thia­diazo­lium-2-yl moiety in a penta­fluorido­arsenate adduct (Roesky *et al.*, 1986 ▸).

**Figure 1:**
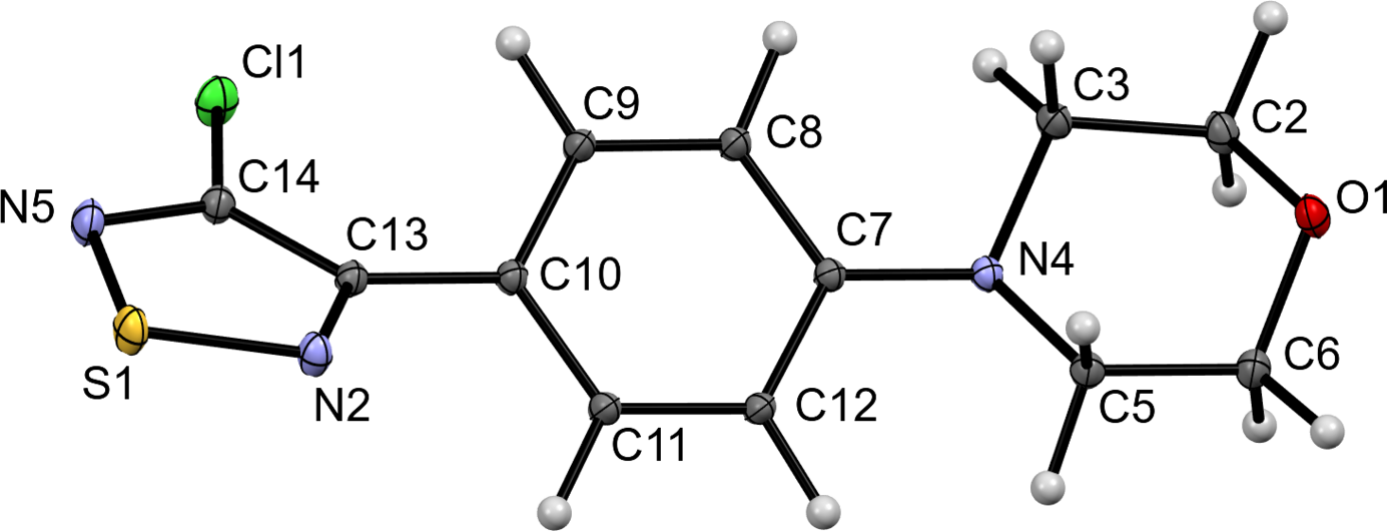
Displacement ellipsoid plot of the title compound (50% probability level). H atoms are shown as small spheres of arbitrary radius.

**Figure 2:**
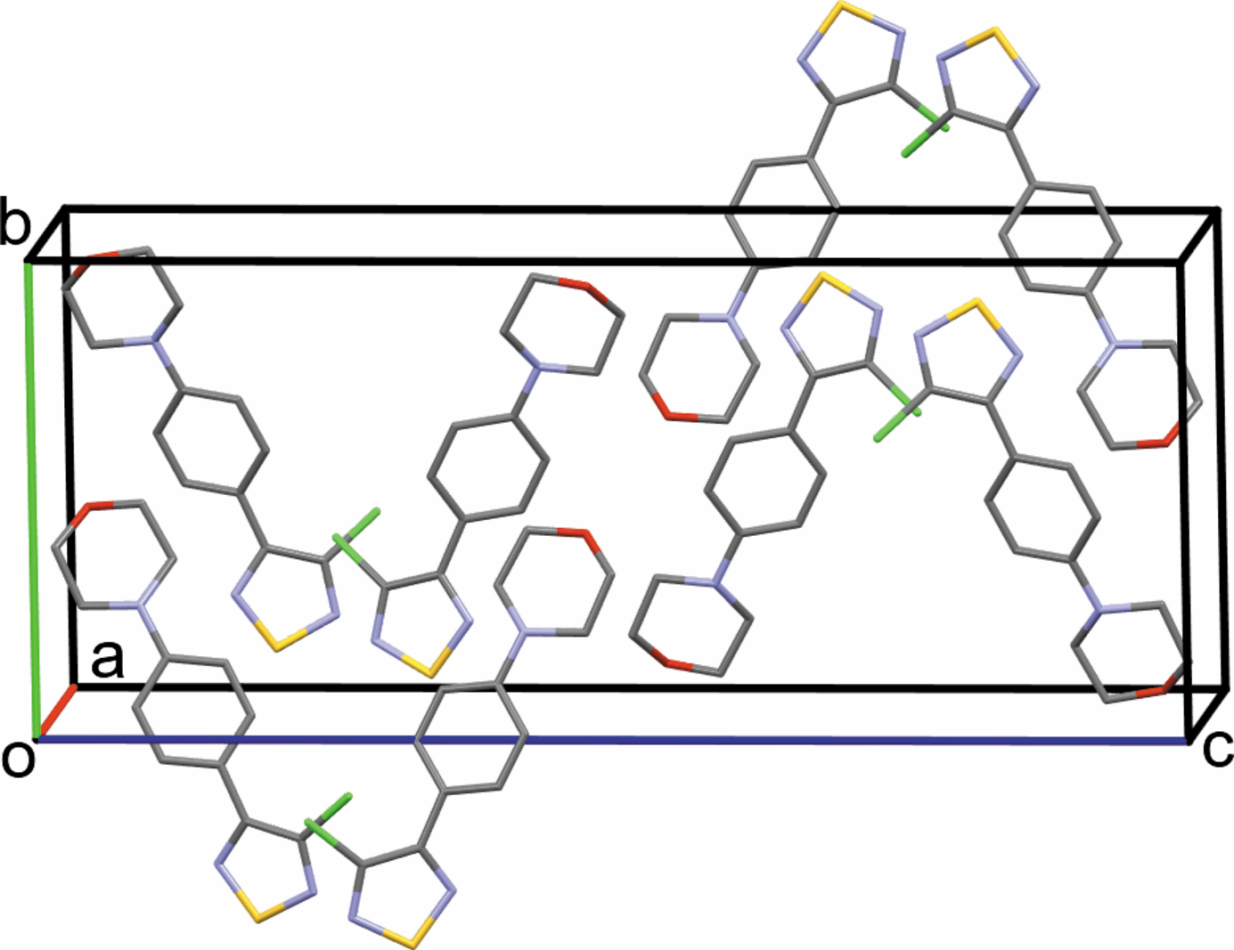
View of the ortho­rhom­bic unit cell of the title compound approximately along the *a*-axis direction. H atoms have been omitted for clarity. Colour scheme: C grey, Cl green, N blue, O red and S yellow.

Fig. 1 ▸ shows the mol­ecular structure of the title compound in the crystal. Table 1 ▸ lists geometric parameters within the 1,2,5-thia­diazole ring. These are comparable to those encountered in the above-mentioned UQOGIT and also resemble those in the structure of the parent 1,2,5-thia­diazole, as determined by electron diffraction in the gas phase (Momany & Bonham, 1964 ▸). As in UQOGIT, the electronegative Cl substituent increases the *ipso* N—C—C angle as compared with the aromatic substituent. The dihedral angle between the mean planes of the 1,2,5-thia­diazole ring and the benzene ring is 36.83 (2)°. The morpholine ring exhibits the expected low-energy chair conformation and is slightly twisted out of the plane of the benzene ring. The bonding situation at the morpholine N atom is markedly pyramidal, as indicated by Σ(C—N—C) = 343.21 (7)°, which is significantly smaller than the value of 360° in the case of a perfectly planar coordination. The pyramidal height, *i.e.* the perpendicular distance from N4 to the plane defined by C3, C5 and C7, is 0.3473 (4) Å. Fig. 2 ▸ depicts the arrangement of the mol­ecules in the ortho­rhom­bic unit cell. A packing index of 75%, as calculated with *PLATON* (Spek, 2020 ▸), reveals a dense crystal packing (Kitajgorodskij, 1973 ▸).

**Figure 3:**
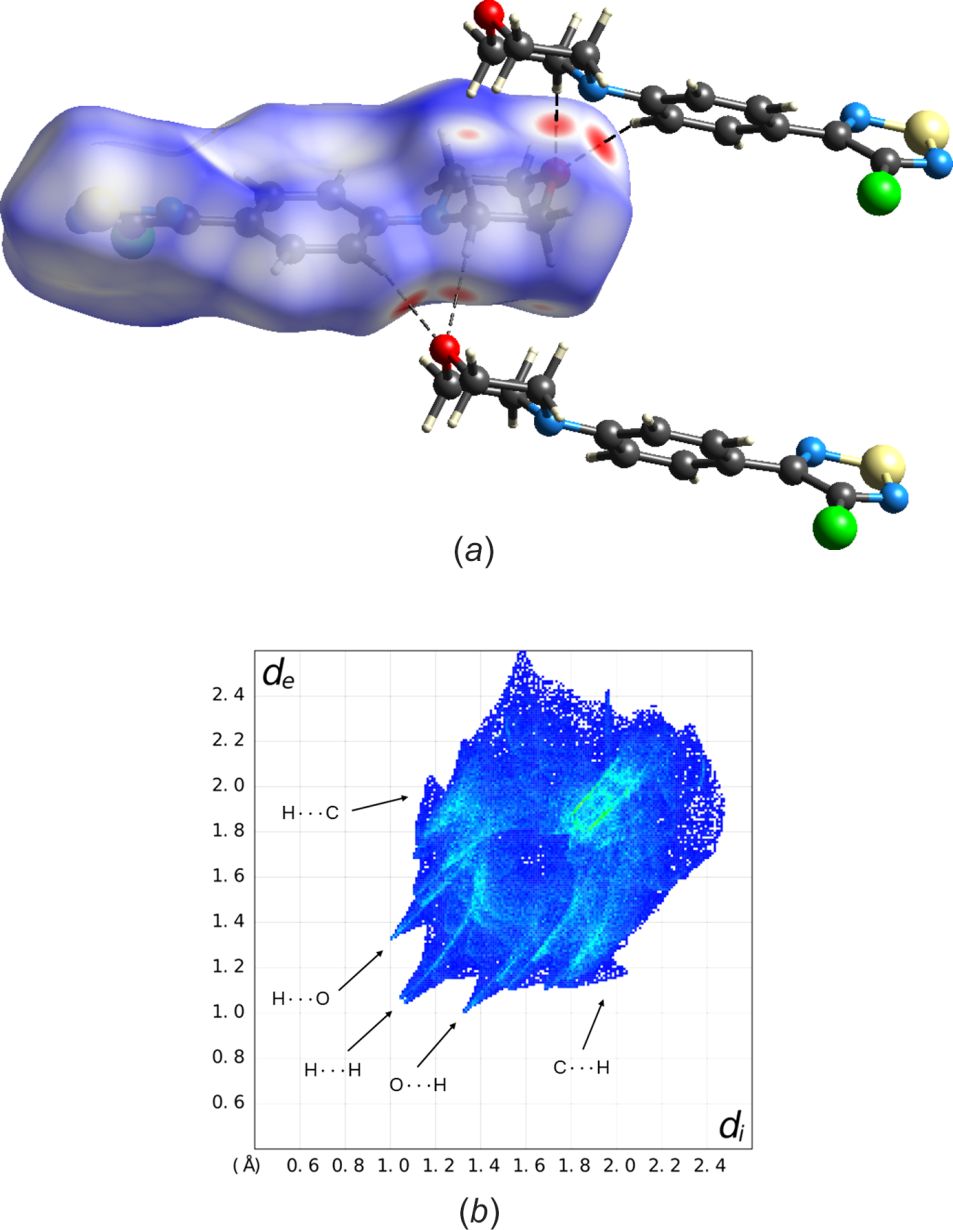
(*a*) Hirshfeld surface mapped with *d*_norm_ for the title compound and (*b*) the corresponding two-dimensional fingerprint plot, where *d*_i_ and *d*_e_ are the distances from a point on the Hirshfeld surface to the nearest atom inside and outside the surface, respectively. Dashed lines represent weak hydrogen bonds. Colour scheme for the atoms: C grey, H white, Cl green, N blue, O red and S yellow.

To better understand the mol­ecular environment of the title compound, we carried out a Hirshfeld surface analysis (Spackman & Jayatilaka, 2009 ▸) using *CrystalExplorer21* (Spackman *et al.*, 2021 ▸). Fig. 3 ▸(*a*) shows the Hirshfeld surface for the title compound mapped with the normalized contact distance (*d*_norm_), with the colours indicating inter­molecular contacts shorter (red), approximately equal (white) or longer (blue) than the sum of the van der Waals radii (Bondi, 1964 ▸). Inspection of the *d*_norm_ plot reveals two large red concave areas associated with the C5—H5*A*⋯O1^ii^ and C12—H12⋯O1^ii^ inter­molecular contacts, which can be regarded as weak hydrogen bonds (Table 2 ▸). A small red area arises from a short inter­molecular H⋯H contact between the morpholine rings of adjacent mol­ecules. In contrast, the H⋯*A* separation in the C3—H3*A*⋯N2^i^ inter­molecular contact (Table 2 ▸) is close to the sum of the corresponding van der Waals radii (bearing in mind that *CrystalExplorer21* by default sets neutron-normalized *X*—H distances; Allen & Bruno, 2010 ▸) and is not associated with a red area in the *d*_norm_ plot. Fig. 3 ▸(*b*) shows the corresponding fingerprint plot. For H⋯H contacts (26.4% contribution of close contacts to the Hirshfeld surface), the tip on the diagonal occurs at *d*_e_ + *d*_i_ < 2.4 Å (*i.e.* less than two times the van der Waals radius of hydrogen) and corresponds to the small red spot in the *d*_norm_ plot in Fig. 3 ▸(*a*). Moverover, the fingerprint plot shows the two spikes for H⋯O/O⋯H contacts (6.4% contribution) from the C—H⋯O weak hydrogen bonds and wings associated with H⋯C/C⋯H contacts (13.8% contribution).

## Synthesis and crystallization

Starting materials were purchased and used as received. NMR spectra were recorded on an Agilent Technologies 400 MHz VNMRS spectrometer. Chemical shifts are reported relative to the residual solvent signal of chloro­form-*d* (δ_H_ = 7.26 ppm, δ_C_ = 77.16 ppm). Abbreviation: *m* = multiplet.

(4-Morpholino­phen­yl)boronic acid (615 mg, 2.97 mmol) was dissolved in toluene (40 ml), 1,4-dioxane (5 ml) and di­methyl­formamide (5 ml) in a 100 ml Schlenk flask. Caesium fluoride (1.83 g, 12.0 mmol) dissolved in approximately 0.5 ml of deionized water, tetra­kis­(tri­phenyl­phosphane)palladium(0) (231 mg, 0.20 mmol) and 3,4-di­chloro-1,2,5-thia­diazole (620 mg, 4.00 mmol) were added under an argon atmosphere. Subsequently, the mixture was heated to 363 K for 12 h with magnetic stirring, whereupon the colour turned from yellow to red. After filtering through Celite, the solvents were removed under reduced pressure co-evaporation using toluene (2 × 20 ml of toluene were added to the residue and evaporated). The crude product was purified by flash chromatography (Inter­chim puriFlash 430) on silica gel using gradient elution (*n*-heptane with ethyl acetate 0 to 40% *v*/*v*) to yield the title compound as a yellow oil (167 mg, 0.59 mmol, 20%). ^1^H NMR (402 MHz, chloro­form-*d*) δ 7.55–7.46 (*m*, 2H), 6.90–6.83 (*m*, 2H), 3.88–3.81 (*m*, 4H), 3.31–3.24 (*m*, 4H) ppm. ^13^C{^1^H} NMR (101 MHz, chloro­form-*d*): δ 153.6, 133.6, 128.5, 120.0, 114.2, 101.1, 66.6, 47.5 ppm. Crystals suitable for X-ray diffraction analysis were obtained when a solution of the compound in chloro­form-*d* was allowed to evaporate slowly under ambient conditions.

## Refinement

Crystal data and refinement details are given in Table 3 ▸. Initial independent atom model (IAM) refinement was carried out with *SHELXL* (Sheldrick, 2015*b* ▸). The final model from IAM refinement was then used as the starting point for Hirshfeld atom refinement using *NoSpherA2* (Kleemiss *et al.*, 2021 ▸) in *OLEX2* (Dolomanov *et al.*, 2009 ▸). Within *NoSpherA2*, *ORCA* (Version 6.1; Neese, 2025 ▸) was used to calculate the electron density at the B3LYP/def2-TZVPP level of theory (Becke, 1993 ▸; Lee *et al.*, 1988 ▸; Weigend & Ahlrichs, 2005 ▸), which was subsequently partitioned into Hirshfeld atoms and converted *via* Fourier transform into atomic form factors (Midgley *et al.*, 2021 ▸). Least-squares refinements against the non-spherical atomic form factors so obtained were performed using *olex2.refine* (Bourhis *et al.*, 2015 ▸). Anisotropic atomic displacement parameters (ADPs) were refined for all non-H atoms. The positions and isotropic ADPs of the H atoms were refined freely.

## Supplementary Material

Crystal structure: contains datablock(s) I, global. DOI: 10.1107/S2414314626004207/zl4097sup1.cif

Structure factors: contains datablock(s) I. DOI: 10.1107/S2414314626004207/zl4097Isup2.hkl

Supporting information file. DOI: 10.1107/S2414314626004207/zl4097Isup3.cdx

1H and 13C NMR spectra of the title compound. DOI: 10.1107/S2414314626004207/zl4097sup3.pdf

Supporting information file. DOI: 10.1107/S2414314626004207/zl4097Isup5.cml

CCDC reference: 2548183

Additional supporting information:  crystallographic information; 3D view; checkCIF report

## Figures and Tables

**Table 1:** Selected geometric parameters (Å, °)

C13—C14	1.4364 (7)	N2—S1	1.6274 (4)
C13—N2	1.3298 (6)	N5—S1	1.6304 (5)
C14—N5	1.3133 (6)		
			
N2—C13—C14	110.85 (4)	S1—N5—C14	106.12 (3)
N5—C14—C13	115.65 (4)	N5—S1—N2	99.09 (2)
S1—N2—C13	108.29 (3)		

**Table 2:** Hydrogen-bond geometry (Å, °)

*D*—H⋯*A*	*D*—H	H⋯*A*	*D*⋯*A*	*D*—H⋯*A*
C3—H3*A*⋯N2^i^	1.107 (7)	2.608 (7)	3.6913 (7)	165.8 (5)
C5—H5*A*⋯O1^ii^	1.070 (7)	2.406 (7)	3.4529 (6)	165.6 (5)
C12—H12⋯O1^ii^	1.063 (6)	2.348 (7)	3.3275 (6)	152.6 (5)

Symmetry codes: (i) 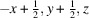; (ii) 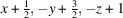.

**Table 3:** Experimental details

Crystal data	
Chemical formula	C_12_H_12_ClN_3_OS
*M*_r_	281.77
Crystal system, space group	Orthorhombic, *Pbca*
Temperature (K)	100
*a*, *b*, *c* (Å)	7.5584 (4), 11.5187 (6), 27.5229 (16)
*V* (Å^3^)	2396.2 (2)
*Z*	8
Radiation type	Mo *K*α
μ (mm^−1^)	0.48
Crystal size (mm)	0.24 × 0.15 × 0.10

Data collection	
Diffractometer	Bruker Kappa Mach3 APEXII
Absorption correction	Gaussian (*SADABS*; Krause *et al.*, 2015 ▸)
*T*_min_, *T*_max_	0.933, 0.963
No. of measured, independent and observed [*I* ≥ 2u(*I*)] reflections	89511, 5236, 4448
*R*_int_	0.040
(sin θ/λ)_max_ (Å^−1^)	0.806

Refinement	
*R*[*F*^2^ > 2σ(*F*^2^)], *wR*(*F*^2^), *S*	0.020, 0.032, 1.04
No. of reflections	5236
No. of parameters	211
H-atom treatment	All H-atom parameters refined
Δρ_max_, Δρ_min_ (e Å^−3^)	0.32, −0.25

Computer programs: *APEX3* (Bruker, 2016 ▸), *SAINT* (Bruker, 2004 ▸), *SHELXT* (Sheldrick, 2015*a* ▸), *olex2.refine* (Bourhis *et al.*, 2015 ▸), *SHELXL* (Sheldrick, 2015*b* ▸), *Mercury* (Macrae *et al.*, 2020 ▸) and *publCIF* (Westrip, 2010 ▸).

## full crystallographic data

### 4-[4-(4-Chloro-1,2,5-thiadiazol-3-yl)phenyl]morpholine Crystal data

**Table:**

C_12_H_12_ClN_3_OS	*D*_x_ = 1.562 Mg m^−^^3^
*M**_r_* = 281.77	Mo *K*α radiation, λ = 0.71073 Å
Orthorhombic, *P**b**c**a*	Cell parameters from 9139 reflections
*a* = 7.5584 (4) Å	θ = 3.0–34.7°
*b* = 11.5187 (6) Å	µ = 0.48 mm^−^^1^
*c* = 27.5229 (16) Å	*T* = 100 K
*V* = 2396.2 (2) Å^3^	Prism, yellow
*Z* = 8	0.24 × 0.15 × 0.10 mm
*F*(000) = 1170.773	

### 4-[4-(4-Chloro-1,2,5-thiadiazol-3-yl)phenyl]morpholine Data collection

**Table:**

Bruker Kappa Mach3 APEXII diffractometer	5236 independent reflections
Radiation source: IµS	4448 reflections with *I*≥ 2u(*I*)
Incoatec Helios mirrors monochromator	*R*_int_ = 0.040
Detector resolution: 66.67 pixels mm^-1^	θ_max_ = 35.0°, θ_min_ = 1.5°
φ– and ω–scans	*h* = −12→12
Absorption correction: gaussian (SADABS; Krause et al, 2015)	*k* = −18→18
*T*_min_ = 0.933, *T*_max_ = 0.963	*l* = −43→44
89511 measured reflections	

### 4-[4-(4-Chloro-1,2,5-thiadiazol-3-yl)phenyl]morpholine Refinement

**Table:**

Refinement on *F*^2^	Primary atom site location: dual
Least-squares matrix: full	Secondary atom site location: difference Fourier map
*R*[*F*^2^ > 2σ(*F*^2^)] = 0.020	Hydrogen site location: difference Fourier map
*wR*(*F*^2^) = 0.032	All H-atom parameters refined
*S* = 1.03	*w* = 1/[σ^2^(*F*_o_^2^) + (0.0074*P*)^2^ + 0.278*P*] where *P* = (*F*_o_^2^ + 2*F*_c_^2^)/3
5236 reflections	(Δ/σ)_max_ = −0.001
211 parameters	Δρ_max_ = 0.32 e Å^−^^3^
0 restraints	Δρ_min_ = −0.25 e Å^−^^3^
0 constraints	

### 4-[4-(4-Chloro-1,2,5-thiadiazol-3-yl)phenyl]morpholine Special details

**Table:**

Experimental. Crystal mounted on a MiTeGen loop using Perfluoropolyether PFO-XR75
Refinement. Refinement using NoSpherA2, an implementation of NOn-SPHERical Atom-form-factors in Olex2. Please cite: F. Kleemiss *et al.* Chem. Sci. DOI 10.1039/D0SC05526C - 2021 NoSpherA2 implementation of HAR makes use of tailor-made aspherical atomic form factors calculated on-the-fly from a Hirshfeld-partitioned electron density (ED) - not from spherical-atom form factors.The ED is calculated from a gaussian basis set single determinant SCF wavefunction - either Hartree-Fock or DFT using selected funtionals - for a fragment of the crystal. This fragment can be embedded in an electrostatic crystal field by employing cluster charges or modelled using implicit solvation models, depending on the software used.

### 4-[4-(4-Chloro-1,2,5-thiadiazol-3-yl)phenyl]morpholine Fractional atomic coordinates and isotropic or equivalent isotropic displacement parameters (Å^2^)

**Table:**

	*x*	*y*	*z*	*U*_iso_*/*U*_eq_	
C2	0.38502 (7)	0.92641 (4)	0.425523 (18)	0.01240 (9)	
H2A	0.5161 (10)	0.9672 (6)	0.4261 (2)	0.0279 (18)*	
H2B	0.2929 (9)	0.9833 (6)	0.4074 (2)	0.0281 (17)*	
C3	0.39295 (7)	0.81117 (4)	0.398916 (19)	0.01214 (9)	
H3A	0.2567 (10)	0.7782 (6)	0.3937 (2)	0.0326 (19)*	
H3B	0.4511 (9)	0.8271 (6)	0.3634 (3)	0.0293 (18)*	
C5	0.45592 (7)	0.71994 (4)	0.476838 (18)	0.01125 (9)	
H5A	0.5541 (9)	0.6714 (6)	0.4962 (2)	0.0286 (18)*	
H5B	0.3287 (9)	0.6744 (6)	0.4811 (2)	0.0281 (18)*	
C6	0.44555 (7)	0.83994 (4)	0.499362 (19)	0.01331 (9)	
H6A	0.3980 (9)	0.8330 (6)	0.5368 (3)	0.0295 (18)*	
H6B	0.5775 (9)	0.8807 (6)	0.4993 (2)	0.0307 (18)*	
C7	0.52410 (6)	0.61898 (4)	0.401643 (17)	0.00858 (8)	
C8	0.48098 (7)	0.60330 (4)	0.352314 (18)	0.01083 (9)	
H8	0.4279 (9)	0.6729 (6)	0.3307 (2)	0.0271 (18)*	
C9	0.50209 (7)	0.49617 (4)	0.329747 (18)	0.01090 (8)	
H9	0.4654 (9)	0.4885 (6)	0.2924 (2)	0.0250 (17)*	
C10	0.56536 (6)	0.39984 (4)	0.355300 (17)	0.00928 (8)	
C11	0.61137 (7)	0.41537 (4)	0.404152 (17)	0.01015 (8)	
H11	0.6638 (9)	0.3425 (6)	0.4246 (2)	0.0246 (17)*	
C12	0.59249 (6)	0.52214 (4)	0.426911 (18)	0.00995 (8)	
H12	0.6311 (9)	0.5284 (5)	0.4639 (2)	0.0238 (16)*	
C13	0.57517 (6)	0.28349 (4)	0.333634 (17)	0.00958 (8)	
C14	0.62143 (7)	0.25191 (4)	0.284770 (18)	0.01116 (8)	
Cl1	0.687353 (18)	0.347856 (10)	0.240584 (5)	0.01629 (3)	
N2	0.53607 (6)	0.18989 (3)	0.359801 (16)	0.01227 (8)	
N4	0.50157 (6)	0.72611 (3)	0.425004 (14)	0.00921 (7)	
N5	0.61572 (6)	0.14042 (4)	0.274917 (16)	0.01455 (8)	
O1	0.32449 (5)	0.91267 (3)	0.474029 (13)	0.01268 (7)	
S1	0.555918 (19)	0.076238 (10)	0.325098 (5)	0.01464 (3)	

### 4-[4-(4-Chloro-1,2,5-thiadiazol-3-yl)phenyl]morpholine Atomic displacement parameters (Å^2^)

**Table:**

	*U*^11^	*U*^22^	*U*^33^	*U*^12^	*U*^13^	*U*^23^
C2	0.0156 (2)	0.00861 (18)	0.0130 (2)	0.00196 (17)	0.00191 (18)	0.00020 (16)
C3	0.0170 (2)	0.00870 (18)	0.0108 (2)	0.00192 (17)	−0.00117 (18)	0.00038 (16)
C5	0.0144 (2)	0.00977 (18)	0.0096 (2)	0.00127 (17)	−0.00004 (18)	−0.00046 (15)
C6	0.0174 (2)	0.01161 (19)	0.0109 (2)	0.00261 (18)	−0.00030 (19)	−0.00234 (17)
C7	0.0102 (2)	0.00721 (17)	0.0084 (2)	−0.00005 (15)	−0.00072 (16)	−0.00013 (15)
C8	0.0161 (2)	0.00774 (18)	0.0086 (2)	0.00140 (16)	−0.00195 (17)	−0.00022 (15)
C9	0.0163 (2)	0.00841 (18)	0.0080 (2)	0.00109 (16)	−0.00197 (17)	−0.00071 (16)
C10	0.0122 (2)	0.00704 (17)	0.0086 (2)	0.00006 (15)	−0.00089 (17)	−0.00057 (14)
C11	0.0140 (2)	0.00743 (17)	0.0091 (2)	0.00062 (16)	−0.00205 (17)	−0.00002 (15)
C12	0.0134 (2)	0.00818 (18)	0.0083 (2)	0.00056 (15)	−0.00264 (17)	−0.00056 (15)
C13	0.0117 (2)	0.00786 (17)	0.0092 (2)	−0.00008 (15)	−0.00026 (16)	−0.00088 (15)
C14	0.0134 (2)	0.00995 (18)	0.0101 (2)	0.00038 (16)	0.00056 (17)	−0.00111 (16)
Cl1	0.02243 (6)	0.01487 (5)	0.01157 (5)	−0.00035 (5)	0.00489 (5)	0.00119 (4)
N2	0.0174 (2)	0.00820 (16)	0.0112 (2)	−0.00033 (15)	0.00177 (16)	−0.00013 (14)
N4	0.01069 (18)	0.00762 (15)	0.00932 (18)	0.00034 (13)	−0.00017 (14)	−0.00057 (13)
N5	0.0202 (2)	0.01077 (17)	0.0126 (2)	0.00121 (16)	0.00077 (17)	−0.00376 (15)
O1	0.01367 (17)	0.01125 (15)	0.01311 (17)	0.00307 (13)	0.00292 (13)	−0.00071 (12)
S1	0.02151 (6)	0.00717 (4)	0.01524 (6)	−0.00030 (4)	0.00130 (5)	−0.00138 (4)

### 4-[4-(4-Chloro-1,2,5-thiadiazol-3-yl)phenyl]morpholine Geometric parameters (Å, º)

**Table:**

C2—H2A	1.096 (7)	C7—N4	1.4019 (6)
C2—H2B	1.078 (7)	C8—H8	1.075 (7)
C2—C3	1.5171 (7)	C8—C9	1.3907 (6)
C2—O1	1.4201 (6)	C9—H9	1.069 (7)
C3—H3A	1.107 (7)	C9—C10	1.3980 (6)
C3—H3B	1.088 (7)	C10—C11	1.4002 (7)
C3—N4	1.4661 (6)	C10—C13	1.4688 (6)
C5—H5A	1.070 (7)	C11—H11	1.085 (6)
C5—H5B	1.101 (7)	C11—C12	1.3875 (6)
C5—C6	1.5169 (7)	C12—H12	1.063 (6)
C5—N4	1.4695 (6)	C13—C14	1.4364 (7)
C6—H6A	1.094 (7)	C13—N2	1.3298 (6)
C6—H6B	1.102 (7)	C14—Cl1	1.7172 (5)
C6—O1	1.4231 (6)	C14—N5	1.3133 (6)
C7—C8	1.4079 (7)	N2—S1	1.6274 (4)
C7—C12	1.4125 (6)	N5—S1	1.6304 (5)
			
H2B—C2—H2A	109.2 (5)	C9—C8—C7	121.20 (4)
C3—C2—H2A	110.3 (4)	C9—C8—H8	117.2 (3)
C3—C2—H2B	109.5 (4)	H9—C9—C8	118.2 (3)
O1—C2—H2A	109.0 (3)	C10—C9—C8	121.25 (4)
O1—C2—H2B	107.1 (3)	C10—C9—H9	120.5 (3)
O1—C2—C3	111.65 (4)	C11—C10—C9	117.81 (4)
H3A—C3—C2	109.0 (4)	C13—C10—C9	122.49 (4)
H3B—C3—C2	107.6 (4)	C13—C10—C11	119.60 (4)
H3B—C3—H3A	108.5 (5)	H11—C11—C10	119.3 (3)
N4—C3—C2	111.74 (4)	C12—C11—C10	121.42 (4)
N4—C3—H3A	110.8 (4)	C12—C11—H11	119.3 (3)
N4—C3—H3B	109.0 (4)	C11—C12—C7	121.02 (4)
H5B—C5—H5A	107.7 (5)	H12—C12—C7	121.3 (3)
C6—C5—H5A	108.0 (4)	H12—C12—C11	117.7 (3)
C6—C5—H5B	110.2 (4)	C14—C13—C10	128.57 (4)
N4—C5—H5A	110.2 (4)	N2—C13—C10	120.58 (4)
N4—C5—H5B	109.4 (4)	N2—C13—C14	110.85 (4)
N4—C5—C6	111.39 (4)	Cl1—C14—C13	124.80 (4)
H6A—C6—C5	109.6 (4)	N5—C14—C13	115.65 (4)
H6B—C6—C5	109.9 (4)	N5—C14—Cl1	119.52 (4)
H6B—C6—H6A	109.2 (5)	S1—N2—C13	108.29 (3)
O1—C6—C5	111.68 (4)	C5—N4—C3	112.10 (4)
O1—C6—H6A	107.1 (4)	C7—N4—C3	115.57 (4)
O1—C6—H6B	109.3 (4)	C7—N4—C5	115.54 (4)
C12—C7—C8	117.27 (4)	S1—N5—C14	106.12 (3)
N4—C7—C8	121.81 (4)	C6—O1—C2	108.60 (4)
N4—C7—C12	120.92 (4)	N5—S1—N2	99.09 (2)
H8—C8—C7	121.6 (3)		
			
C2—C3—N4—C5	48.09 (5)	C9—C10—C13—C14	37.71 (6)
C2—C3—N4—C7	−176.66 (4)	C9—C10—C13—N2	−141.29 (5)
C2—O1—C6—C5	−61.73 (4)	C10—C13—C14—Cl1	3.27 (6)
C3—C2—O1—C6	61.40 (5)	C10—C13—C14—N5	−178.58 (5)
C3—N4—C5—C6	−48.20 (4)	C10—C13—N2—S1	178.84 (4)
C3—N4—C7—C8	14.02 (5)	C11—C10—C13—C14	−145.91 (5)
C3—N4—C7—C12	−166.35 (4)	C11—C10—C13—N2	35.09 (5)
C5—N4—C7—C8	147.73 (4)	C11—C12—C7—N4	178.61 (4)
C5—N4—C7—C12	−32.64 (5)	C12—C11—C10—C13	−175.54 (5)
C6—C5—N4—C7	176.53 (4)	C13—C14—N5—S1	−0.41 (4)
C7—C8—C9—C10	0.59 (6)	C13—N2—S1—N5	0.09 (4)
C7—C12—C11—C10	0.70 (6)	C14—C13—N2—S1	−0.32 (4)
C8—C7—C12—C11	−1.74 (5)	C14—N5—S1—N2	0.19 (4)
C8—C9—C10—C11	−1.65 (6)	Cl1—C14—C13—N2	−177.66 (4)
C8—C9—C10—C13	174.79 (5)	Cl1—C14—N5—S1	177.85 (4)
C9—C8—C7—C12	1.11 (6)	N2—C13—C14—N5	0.50 (5)
C9—C8—C7—N4	−179.25 (5)	N4—C3—C2—O1	−55.33 (5)
C9—C10—C11—C12	1.01 (5)	N4—C5—C6—O1	55.67 (4)

### 4-[4-(4-Chloro-1,2,5-thiadiazol-3-yl)phenyl]morpholine Hydrogen-bond geometry (Å, º)

**Table:**

*D*—H···*A*	*D*—H	H···*A*	*D*···*A*	*D*—H···*A*
C3—H3*A*···N2^i^	1.107 (7)	2.608 (7)	3.6913 (7)	165.8 (5)
C5—H5*A*···O1^ii^	1.070 (7)	2.406 (7)	3.4529 (6)	165.6 (5)
C12—H12···O1^ii^	1.063 (6)	2.348 (7)	3.3275 (6)	152.6 (5)

Symmetry codes: (i) −*x*+1/2, *y*+1/2, *z*; (ii) *x*+1/2, −*y*+3/2, −*z*+1.
