# Supplementary figures and images for: 4-[4-(4-Chloro-1,2,5-thia­diazol-3-yl)phen­yl]morpholine
Source: IUCrdata. 2026 May 7;11(Pt 5):x260420. doi: 10.1107/S2414314626004207 (PMC13239004; doi:10.1107/S2414314626004207)

$^1\text{H}$  NMR (402 MHz,  $\text{cdCl}_3$ )  $\delta$  7.55 – 7.46 (m, 2H), 6.90 – 6.83 (m, 2H), 3.88 – 3.81 (m, 4H), 3.31 – 3.24 (m, 4H).

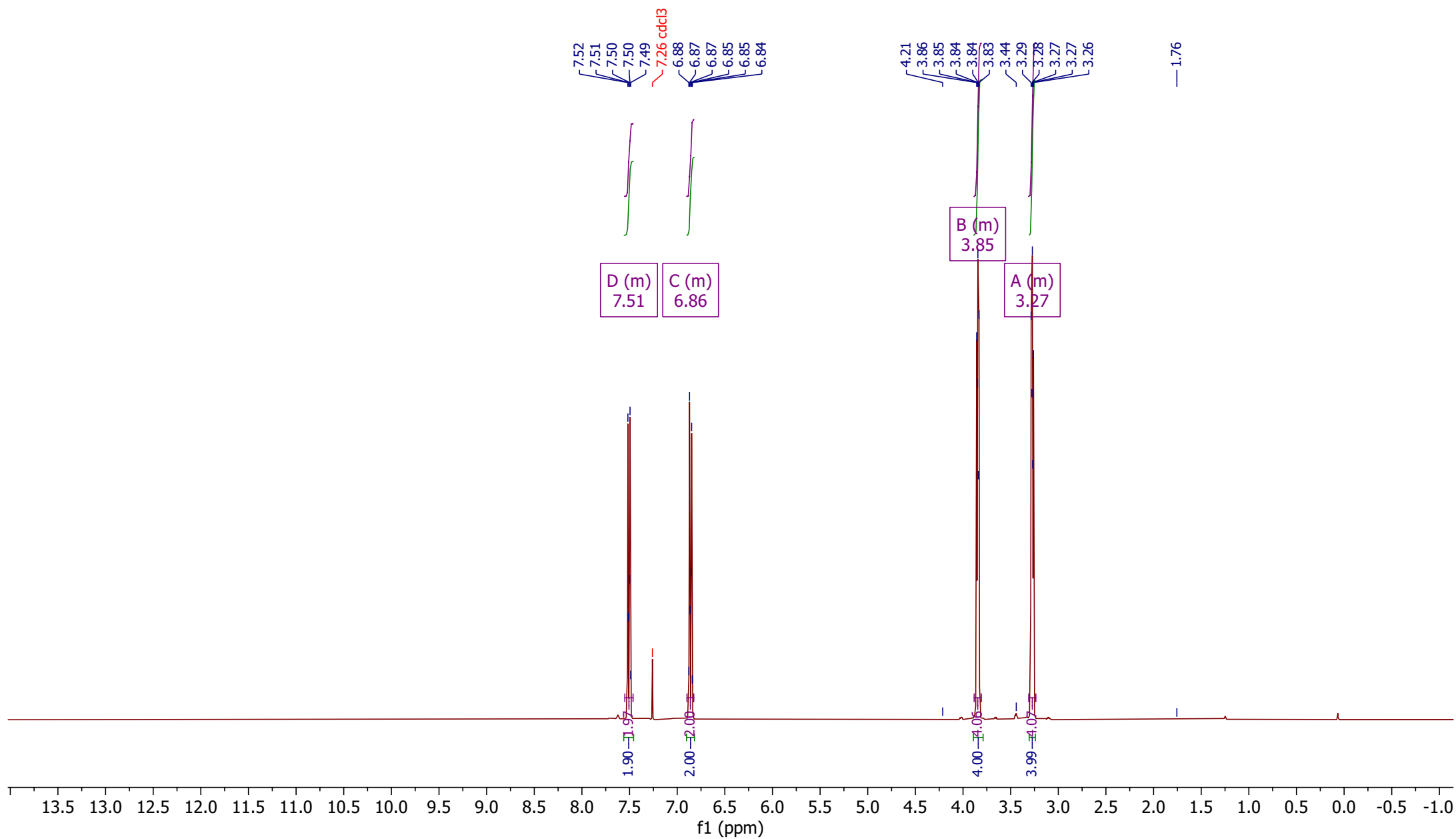

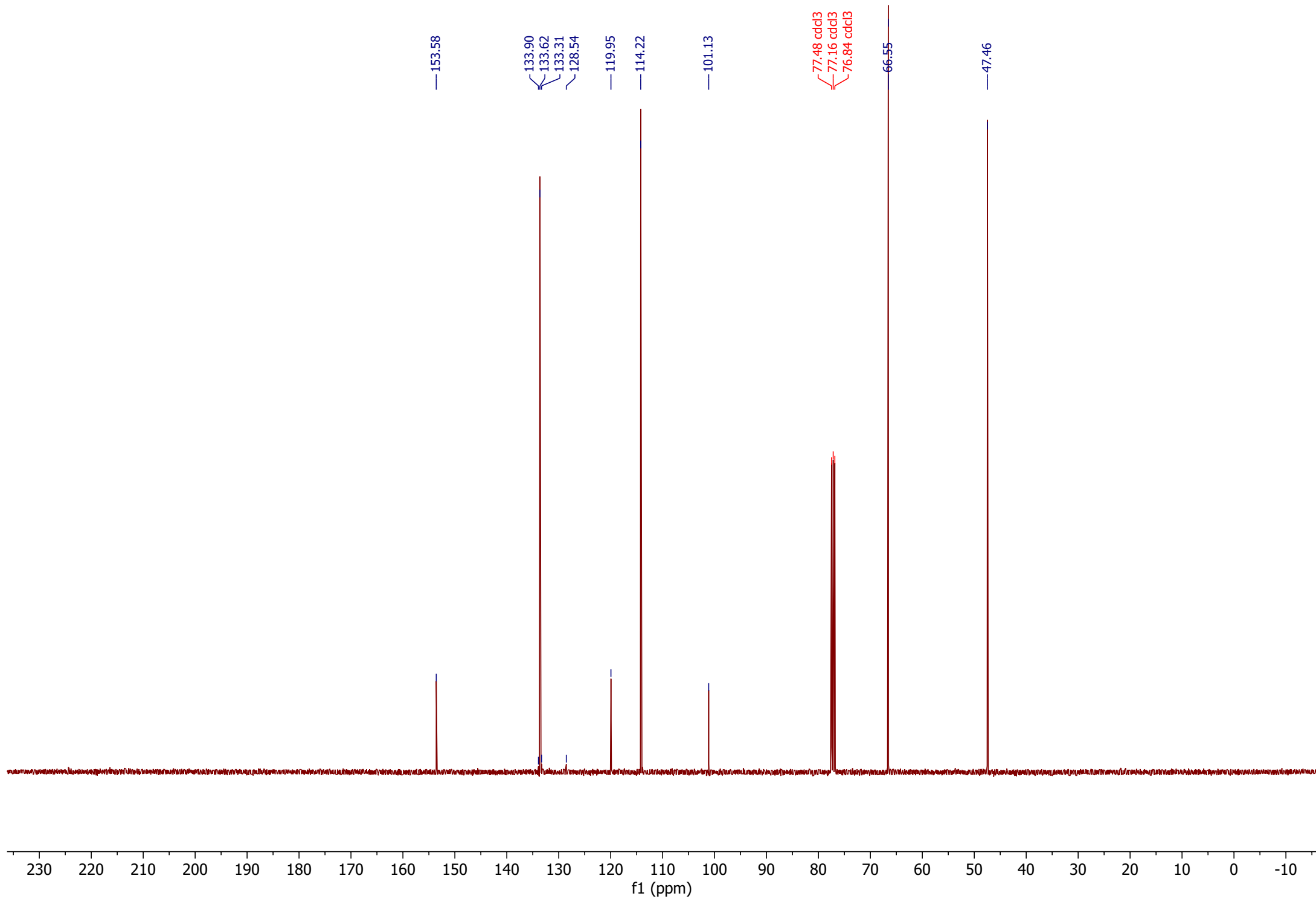

Supplement: Supplementary file 4 [file x-11-x260420-sup3.pdf]
